# Efficacy of integrating a semi-immersive virtual device in the HABIT-ILE intervention for children with unilateral cerebral palsy: a non-inferiority randomized controlled trial

**DOI:** 10.1186/s12984-023-01218-4

**Published:** 2023-07-29

**Authors:** G. Saussez, R. Bailly, R. Araneda, J. Paradis, D. Ebner-Karestinos, A. Klöcker, E. S. Sogbossi, I. Riquelme, S. Brochard, Y. Bleyenheuft

**Affiliations:** 1 UCLouvain, Institute of Neuroscience, COSY Pole, MSL-IN Lab, Brussels, Belgium; 2 Motor Sciences department, FfH Lab, CeREF Santé, HELHa, Rue Trieu Kaisin, 136, 6061 Montignies-Sur-Sambre, Belgium; 3 Fondation Ildys, Brest, France; 4 Laboratoire de Traitement de l’information Médicale (LaTIM), Inserm U1101, Université Bretagne Occidentale, Brest, France; 5 Exercise and Rehabilitation Science Institute, School of Physical Therapy, Faculty of Rehabilitation Science, Universidad Andres Bello, Santiago, Chile; 6 Department of Developmental Neuroscience, IRCCS Fondazione Stella Maris, Pisa, Italy; 7 Haute Ecole Leonard de Vinci, Parnasse-ISEI, Brussels, Belgium; 8 School of Physical Therapy, Faculty of Health Sciences, University of Abomey-Calavi, Cotonou, Benin; 9 Research Institute of Health Sciences (IUNICS-IdISBa), University of the Balearic Islands, Palma, Spain; 10 Department of Nursing and Physiotherapy, University of the Balearic Islands, Palma, Spain

**Keywords:** Cerebral palsy, Virtual reality, Motor skill learning, Motor function, HABIT-ILE, Rehabilitation, Active video game

## Abstract

**Background:**

The implementation of virtual devices can facilitate the role of therapists (e.g., patient motivation, intensity of practice) to improve the effectiveness of treatment for children with cerebral palsy. Among existing therapeutic devices, none has been specifically designed to promote the application of principles underlying evidence-based motor skill learning interventions. Consequently, evidence is lacking regarding the effectiveness of virtual-based sessions in motor function rehabilitation with respect to promoting the transfer of motor improvements into daily life activities. We tested the effectiveness of implementing a recently developed virtual device (REAtouch^®^), specifically designed to enable the application of therapeutic motor skill learning principles, during a Hand Arm Bimanual Intensive Therapy Including Lower Extremities (HABIT-ILE) intervention.

**Methods:**

Forty children with unilateral cerebral palsy (5–18 years; MACS I-III; GMFCS I-II) were randomly assigned to a control group or a “REAtouch^®^” experimental group for a 90-h HABIT-ILE day-camp intervention (two weeks). Children in the REAtouch^®^ group spent nearly half of their one-on-one therapeutic time using the REAtouch^®^. Participants underwent three testing sessions: the week before (T1), after intervention (T2), and at three months follow-up (T3). The primary outcome was the Assisting Hand Assessment (T3–T1; blinded). Secondary outcomes measured uni-bimanual hand function, stereognosis, gait endurance, daily life abilities, and functional goals. Accelerometers and a manual report of daily activities served to document therapeutic dosage and treatment characteristics. We used one-way RMANOVA to compare the efficacies of the two interventions, and non-inferiority analyses to contrast changes in the “REAtouch^®^” group versus the “HABIT-ILE” control group.

**Results:**

We found significant improvements in both groups for most of the outcome measures (p < 0.05). There was significant non-inferiority of changes in the REAtouch^®^ group for upper extremities motor function, functional goals attainment, and abilities in daily life activities (p < 0.05).

**Conclusions:**

Use of the REAtouch^®^ device during HABIT-ILE showed non-inferior efficacy compared to the conventional evidence-based HABIT-ILE intervention in children with unilateral cerebral palsy. This study demonstrates the feasibility of using this virtual device in a high dosage camp model, and establishes the possibility of applying the therapeutic principles of motor skill learning during specifically designed virtual-based sessions.

*Trial registration*: Trial registration number: NCT03930836-Registration date on the International Clinical Trials Registry Platform (ICTRP): June 21th, 2018; Registration date on NIH Clinical Trials Registry: April 29th, 2019. First patient enrollment: July 3rd, 2018.

**Supplementary Information:**

The online version contains supplementary material available at 10.1186/s12984-023-01218-4.

## Introduction

Cerebral palsy (CP) is the most common sensorimotor disorder in the pediatric population, with an occurrence ranging from 1.5 to over 3 per 1000 births [1, 2]. Among the large variety of motor function interventions proposed for children with CP, a recently updated systematic review substantiated the efficacy of high dosage intensive training applying the principles of motor skill learning [3]. Such interventions entail intensive and structured practice of play/fun activities with incrementally increasing difficulty, focusing on the attainment of self-determined goals through self-generated movements in a child-friendly environment (e.g., Constraint-Induced Movement Therapy [CIMT] [4], Hand-Arm Bimanual Intensive Therapy [HABIT] [5], Hand-Arm Bimanual Intensive Therapy Including Lower Extremities [HABIT-ILE] [6]). Implementation of motor skill learning approaches has emerged as a therapeutic strategy showing efficacy in improving hand function, with transfer into daily life activities and participation in children with unilateral and bilateral CP [3, 7–9].

Despite these promising results, motor skill learning interventions and their underlying therapeutic principles are rarely applied in the current rehabilitation practice of children with CP [3]. One barrier for their broader application might be the prevailing policy of health insurance providers in many countries, which rarely undertake reimbursement of such high therapeutic dosage modalities, despite their proven efficacy. Another logistic barrier is the lack of training and knowledge among clinicians about how best to apply those relatively new interventions in their current practice [10]. To circumvent these obstacles, the use of cost-effective, well-designed virtual devices as tools for rehabilitation might be of great interest [3]. Such tools could positively influence the child’s motivation, increase the duration, intensity, and frequency of practice sessions, and promote the use of both the more and the less-affected limbs; this approach could also enable therapists to place a greater focus on promoting goal-directed training based on the principles of motor skill learning [11, 12].

Virtual environments/devices can be defined as “computer hardware and software systems generating simulations of real or imagined environments with which participants interact using their own movements” [13, 14]. The virtual environments that are currently available for rehabilitation differ with respect to the device used, the level of immersion, and type of interactions [15]. Unfortunately, the broader use of such devices among therapists is currently reportedly impeded by multiple barriers [16]. To be effective in rehabilitation and amenable for use in clinical practice, virtual devices should meet the needs of clinicians and patients, thus calling for an adequate balance between high adaptability and the risk of overwhelming clinicians with additional decision-making requirements [16].

Research studies on virtual devices have hitherto shown no clear evidence for greater efficacy in hand/arm function than conventional care in children with CP, with generally poorer results seen for commercially “off the shelf” devices compared to engineer-built environments, and no evidence for transfer of the improved motor functions to daily life activities [13, 17–20]. Additionally, literature on virtual-based interventions highlights a key disadvantage of the existing virtual environments (e.g., direct body tracking, hand controllers, head mounted system, robotics), namely an insufficient fidelity of the environment with respect to sensory-motor information (e.g., haptic feedback), spatio-temporal organization (e.g., 3D immersion, depth cues), and objects or environment properties/interactions [13, 21–25]. As such, the evidence is inconclusive for transfer of motor learning from virtual to real environments and real-world situations. Based on this prior evidence in neurorehabilitation, future virtual environments designed for rehabilitation should focus on improving fidelity of the trained environment and obtaining better integration of the motor skill learning principles, as applied in the highlighted evidence-based interventions [3, 21, 26, 27].

The past ten years have seen an increasing number of virtual environments designed for rehabilitation that employ interactions with tangible real objects manipulations. Granted that manipulation of real objects manipulation betters respect environment fidelity, such devices have promoted relatively limited integration of motor skill learning therapeutic principles, which are also lacking in available commercial tools [28–30]. Furthermore, none of the hitherto described devices have yet been tested in well-designed, randomized controlled trials, with comparison to an established, evidence-based motor function intervention. Thus, we feel there is insufficient proof of the efficacy of virtual environment activities in improving motor function, with transfer to daily life activities.

The goal of this study was to test for non-inferiority of improvements in motor function and transfer to daily life activities of children with unilateral CP by replacing half of the therapeutic time of an evidence-based HABIT-ILE high dosage intervention camp with training in REAtouch^®^-based sessions. REAtouch^®^ is a new virtual device designed to provide an environment that facilitates therapist decision-making about how to structure the intervention to target the application of motor skill learning principles. We hypothesized that the use of the REAtouch^®^ device for half of the one-on-one therapeutic time would prove feasible, and would present similar treatment characteristics compared to a usual HABIT-ILE camp. We furthermore predicted non-inferior improvements in motor function and daily life abilities for children with augmentation of their intervention through REAtouch^®^, as compared to children following the usual HABIT-ILE intervention without virtual-based sessions.

## Methods

This project was conducted by the Motor Skill Learning and Intensive Neurorehabilitation (MSL-IN) lab, UCLouvain in Brussels, Belgium, in collaboration with the BEaCHILD team (Latim INSERM UMR) in Brest, France. The protocol was registered on ClinicalTrials.gov (NCT03930836; first submission: June 21th, 2018; first child enrollment, July 03th, 2018) with approval from the human research ethics committee of the Université catholique de Louvain (2013/01MAR/069; Belgian registry number: B403201316810).

Forty children with unilateral CP took part in one of the four HABIT-ILE camps organized over the summers 2018 and 2019, with a common supervisor present at every camp. Children and families first received information about the intervention at reference CP centers, from their physician, or through social media. All children and families received specific information about the HABIT-ILE intervention and the study protocol through emails, phone calls, or face-to-face meetings, and all signed a written, voluntary informed consent before their inclusion in the study protocol. As with the previous trial testing the efficacy of HABT-ILE in children with unilateral CP [7], the inclusion criteria were (1) a diagnosis of unilateral CP, (2) age between 5 and 18 years, (3) possessing the ability to grasp/hold light objects with the more-affected hand, and (4) sufficient cognitive ability to engage in structured games, follow instructions, and complete testing. The exclusion criteria were (1) uncontrolled seizures, (2) botulinum-toxin injection or orthopedic surgery planned six months before the camp or during the study protocol, and (3) possibility of interference in treatment/testing because of uncorrected visual problems. Participants were recruited in four cohorts (two of eight participants in 2018, two of 12 participants in 2019). For each cohort, after providing consent to participate, the children were matched into groups stratified by age, MACS, and GMFCS level. They were randomized to either REAtouch^®^ or HABIT-ILE group using a randomizer website (https://www.randomizer.org/). Participant characteristics are displayed in Table 1.

**Table 1:** Participants characteristics and baseline measures

	REAtouch^®^	HABIT-ILE	T-test; Mann-Whitney^a^
Gender			
Male	10	10	
Female	10	10	
Mean age ± SD (yrs.mo)	9.0 ± 3.1	9.1 ± 2.9	0.55^a^
Dominant hand			
Right	7	8	
Left	13	12	
MACS			
1	4	3	
2	10	11	
3	6	6	
GMFCS			
1	11	12	
2	9	8	
Baseline mean ± SD			
AHA (AHA-units)	54.9 ± 18	58.3 ± 16	0.55
BBT-MA (n)	20.8 ± 13	22 ± 11	0.76
BBT-LA (n)	45.2 ± 13	43.7 ± 15	0.75
JTTHF-MA hand (s)	419 ± 358	412 ± 344	0.87^a^
JTTHF-LA hand (s)	64.6 ± 51	59.6 ± 48	0.44^a^
MFPT-MA hand (n)	6.7 ± 3	6.1 ± 3	0.73^a^
MFPT-LA hand (n)	9.6 ± 0.7	9.4 ± 0.7	0.22^a^
6MWT (meters)	467 ± 90	478 ± 106	0.74
ABILHAND-Kids (logits)	1.66 ± 1.8	1.21 ± 1.4	0.41
ACTIVLIM-CP (logits)	1.9 ± 1.3	1.81 ± 1.1	0.96^a^
PEDI (self-care) (/63)	46.5 ± 10	47.3 ± 10	0.80
ABILOCO-Kids (logits)	2.72 ± 1.7	3.21 ± 1.8	0.40
COPM perf (/10)	3.16 ± 1.3	2.76 ± 0.9	0.54^a^
COPM sat (/10)	3.08 ± 1.2	2.96 ± 1.2	0.55^a^

*MACS* Manual Ability Classification System, *GMFCS* Gross Motor Function Classification System, *MA* more-affected, *LA* less-affected, *AHA* Assisting Hand Assessment, *BBT* Box and Blocks Test, *JTTHF* Jebsen-Taylor Test of Hand Function, *MFPT* Manual Form Perception Test, *6MWT *6 Minutes Walk Test, *PEDI *Pediatric Evaluation of Disability Inventory, *COPM *Canadian Occupational Performance Measure, *perf* performance measure, *sat* satisfaction measure

^a^Mann–Whitney rank sum test

### *The REAtouch*^*®*^* device*

**Fig. 1:**
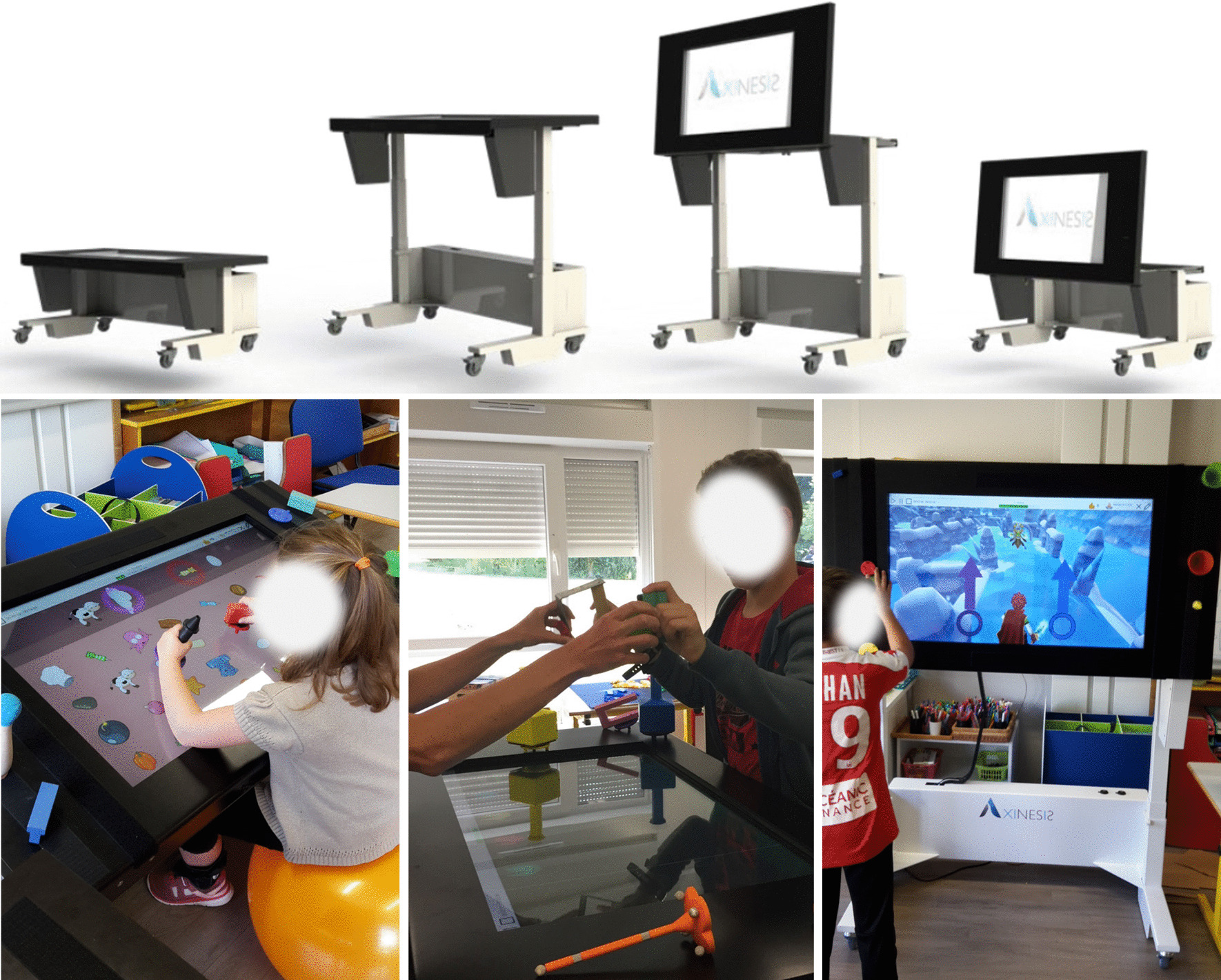
The REAtouch^®^ device. Upper part. 3D modeling of the REAtouch^®^ device. Lower part. Illustration of REAtouch^®^-based sessions during a HABIT-ILE intervention in children with unilateral CP

The REAtouch^®^ has been developed by Axinesis SA in collaboration with expert therapists who provided clinical expertise and guided the conception of the device, its therapeutic content/use, and the approach to promoting the application of efficient motor function rehabilitation (Fig. 1).

**Fig. 2:**
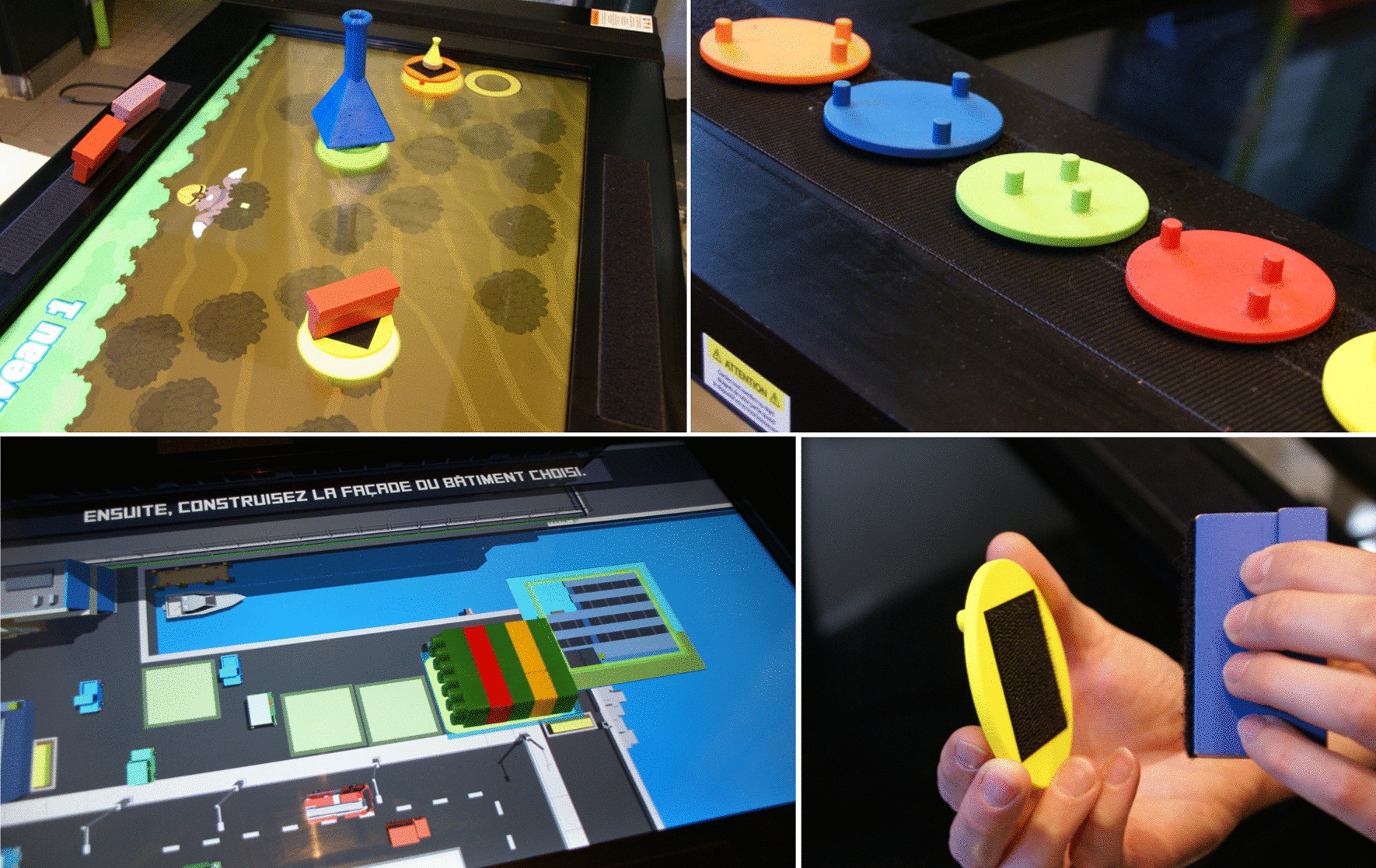
Screen-object interactions, dedicated bases, and Velcro fastening. Upper left panel shows a game using the dedicated bases. The game displayed in the lower left panel shows a game using simple object-screen interactions to construct buildings. The right panels illustrate the five dedicated bases, and the assembly of an object with the yellow base

The REAtouch^®^ is a virtual device designed to provide a visual environment that facilitate therapist decision-making about how best to structure the intervention to target the application of motor skill learning principles: intensity (high dosage and repetitive practice), goal-directed, shaping (training at the just-right level, with incrementally increasing difficulty of the proposed activities), hands-off (self-generated movements driven through adjustment of the affordances of the therapeutic environment), motivation, and feedback on motor task performance [21, 26, 27]. It consists of a 45-in. reactive screen mounted on an adjustable frame allowing the therapist to modulate the height (from 55 to 121 cm) and angle of tilt (from 0° to 85°) of the reactive surface, as required by the particular task. Participants are engaged in a variety of games and activities promoting bimanual repetitive task practice in a personalized game session that integrates reward with challenging and adaptable games. Depending on the games and activities performed, interactions with the reactive surface are made using five dedicated “bases” or simple contact interactions using hands or tangible objects with color matching to the target bases (Fig. 2). As each base is equipped with a Velcro strip, the objects to be manipulated are adaptable to each patient’s motor ability and functional goals by fixing in place the appropriate therapeutically useful object on the dedicated base. More information about the REAtouch^®^ device and its use in this study are presented in Additional files 1 (REAtouch^®^ description and use in theHABIT-ILE context) and 2 (movie illustrating the use of REAtouch^®^ during HABIT-ILE sessions).

### Interventions

All participating children took part in a HABIT-ILE [6] intervention in a high dosage day-camp setting on 10–12 consecutive weekdays, to a total of 90 h. HABIT-ILE is an evidence-based intervention applying motor skill learning principles and focusing on bimanual coordination, postural control, and stimulation of the lower extremities [6]. The learning principles include intensive practice (high therapeutic dosage and motor engagement time) of game/play activities focusing on personalized, self-determined goals so as to maximize motivation and goal-directed training. The training is of tailored specificity, with incrementally increasing difficulty of the proposed tasks (shaping) through self-generated movement using affordances of the environment to provide opportunities to find the optimal strategies (“hands off”) and feedback on motor task performance [6, 26, 27]. The intervention is performed in a child-friendly environment with positive reinforcement from the therapist [6, 21, 26, 27]. At least one interventionist was assigned to each child for the whole therapeutic time, along with trained expert supervision [6]. During HABIT-ILE interventions, the proposed activities with progressive level of difficulty were in three subtypes of activities: (1) table activities, (2) daily living activities, and (3) gross motor play/physical activities (6).

1. For the upper extremities, depending on the child’s motor abilities and functional goals, bimanual activities were progressively graded from a passive support towards more complex/skilled bimanual activities, with adjustments of spatial or temporal constraints and an increasing demand for manipulation and grasping types [6, 7]. For trunk control and lower extremities, activities were performed in different positions, still depending on the child’s ability and functional goals. Table activities were performed while sitting on a bench, or on a plastic ball of varying inflation level, or while standing on an even floor or on a balance board.
2. Daily living activities, including the training of functional goals, can be performed in different positions and settings, with initial learning of a specific technique in an easy/comfortable position, before proceeding to train in a more challenging situation or posture. For example, the child may first learn to tie a shoe placed on a table while sitting in a comfortable position, before proceeding to tie a shoe worn on their foot.
3. Gross motor and physical activities consist mainly of outdoor activities that involve the coordination of both the upper and lower extremities (e.g., cycling, throwing balls, nordic walking, and jumping rope)

For children in the HABIT-ILE group, this protocol was performed using regular society/building/manipulative games and outdoor activities (“usual” HABIT-ILE) [6, 7].

For children randomized in the REAtouch^®^ intervention group, HABIT-ILE was provided through the use of the REAtouch^®^ device for half of the one-on-one therapeutic time (excluding approximately 45 min for lunchtime and 30 min of group activity each day). The planned hours for REAtouch^®^-based sessions entailed about 37 h of a total of 90 h (41%). We chose this proportion of the one-to-one therapeutic time so as to performing REAtouch^®^-based sessions at sufficient dosage to highlight possible inferiority, should thus be the case, while keeping the dosage within the bounds of adherence of children and therapists. We divided the REAtouch^®^ therapeutic time in two sessions (morning and afternoon), each lasting from 90 to 120 min. Although the guiding principle was for an application in motor skill learning, the REAtouch^®^ is properly considered a tool for rehabilitation rather than rehabilitation per se. During REAtouch^®^ based sessions, the regular games and activities (e.g., board games, card, building activities, etc.) are all proposed through the screen of the REAtouch^®^ that interacts with tangible objects in the foreground. These tangible objects are chosen by the therapist to fit the needs of the child and HABIT-ILE requirements. The therapist plays a crucial role in choosing the type of objects to be manipulated, shaping the therapeutic environments, giving appropriate feedback and reinforcement, and promoting the transfer of learned skills to daily activities. All REAtouch^®^-based sessions were directed by an interventionist following the HABIT-ILE principles. While the performance of outdoor activities and some of the functional goals might not always be possible during REAtouch^®^-based sessions, the aim of REAtouch^®^-based sessions is to follow HABIT-ILE therapeutic principles by promoting an intensive and specific practice of the targeted motor abilities. More details about the use of REAtouch^®^ during HABIT-ILE sessions are presented in Additional files 1 and 2.

### Outcome measures

Children had three testing sessions: at baseline, the week before intervention (T1), after intervention (T2), and at three months follow-up (T3). The primary outcome was the Assisting Hand Assessment [31, 32] (AHA; T3-T1). Sessions were video recorded and blindly scored by three trained examiners. Secondary outcomes were the assessment of hand function with the Jebsen-Taylor test of Hand Function [33] (JTTHF) and the Box and Blocks test (BBT) [34]. The Manual Form Perception Test (MFPT) as modified by Cooper et al. [35] assessed stereognosis. Walking endurance was tested using the 6-Minutes-Walk Test [36] (6MWT), and we used parent-reported questionnaires to evaluate the child’s performance and transfer of learning to daily life activities requiring the use of upper extremities (ABILHAND-Kids [37, 38]), locomotor abilities (ABILOCO-Kids [39]), and the coordination of both the upper and lower extremities (ACTIVLIM-CP [40, 41]; Pediatric Evaluation of Disability Inventory [42] PEDI). For the PEDI questionnaire, we used only the self-care subscale functional ability domain. Self-determined functional goals were set at T1 using the Canadian Occupational Performance Measure [43] (COPM). Performance and satisfaction measures were scored on every testing session. These outcome measures are very similar to those used in previous trials showing the efficacy of HABIT-ILE [7, 44, 45].

We collected accelerometer data to control the intensity of intervention in terms of upper extremity movement repetitions. As we had only two accelerometer devices, data were collected for one day for each child, with measures registered on two paired participants during the same day. Accelerometer data were never collected during the first and last days of camps, thus avoiding the potential biases due to novelty at start and unusual camp activities the last day. To further quantify the motor engagement time during the therapeutic intervention and to record the type of activities performed, interventionists systematically reported treatment content, defining the duration and type of activities for upper (gross/fine dexterity, card games, art and craft, functional/daily life activities) and lower (sitting on ball/bench, standing, balance board, walking, running/jumping, bicycle, scooter) extremities. Accelerometer data were collected using a wGT3X-BT monitor (Actigraph, Pensacola, Florida) worn on the wrist of the more-affected upper arm. Wear sensor analysis and manual reporting of treatment schedule were used for wear time validation. Acceleration data were recorded at 30 Hz, downloaded using ActiLife 6.13.4 software (Actigraph, Pensacola, Florida), resampled to 1 Hz, and converted to vector magnitude (VM; square root of the sum of the squares of each axis) activity counts. Activity counts provide an index of frequency (Hz) and intensity (m/s^2^) of the raw acceleration of the limb at a given time point: the higher the counts, the greater the intensity [46, 47]. The affected upper limb activity was quantified by the active duration and the mean VM activity counts. The active duration consists of the percentage time with any registered activity of the more-affected upper limb over the entire monitoring period (i.e., VM > 0). The mean activity count reports the mean VM activity counts per second over the entire monitoring period.

### Statistical analyses

We used the AHA for sample size calculation (α = 0.05, 1 − β = 0.8, δ = 0), which indicated a minimum requirement of 16 participants per group. Considering the likelihood of dropout, we set the sample size to 20 children per group. Baseline outcome measures, accelerometry data, and reported treatment activities were analyzed using T-test or Mann–Whitney comparisons. The effects of both interventions relative to T1 were tested using one-way RMANOVA (or Friedman RMANOVA).

Changes observed between testing sessions in both groups (T2–T1 and T3–T1) were compared using non-inferiority statistics analyses [48, 49]. Non-inferiority statistical analyses are used to test the non-inferiority of the efficacy of the new “alternative approach” (REAtouch^®^ group) compared to a “gold standard” (usual HABIT-ILE group), as distinct from the usually performed superiority statistical analyses that is often used to highlight a difference between groups. To test non-inferiority, one must determine an accepted difference between the new approach and the gold standard («equivalence margin»), which is defined based on an expert’s decision, or as the percentage of the difference between efficacy of the gold-standard and a placebo intervention. As the difference between a potential placebo and the gold-standard is not known for the whole set of outcomes [7], we used the minimal clinically important difference (MCID) as an equivalence margin. MCID values were as previously published (JTTHF [50], BBT [50], MFPT [35], COPM [43]), or were calculated from published data as half of the standard deviation of the observed change [51] (6MWT [7], ABILHAND-Kids [45], ACTIVLIM-CP [41], ABILOCO-Kids [7], PEDI self-care subscale [45]). For the AHA, we used the reported smallest detectable difference of 5 AHA-units, which is considered to represent a true change, exceeding the error variance within the measure [52, 53]. For non-inferiority analyses, the difference between changes observed in the “alternative approach” was compared to that for the “gold standard”, less the equivalence margin set, using T-tests or Mann–Whitney rank sum test. An approach based on one-sided confidence intervals can also be used with a significant non-inferiority being registered if the lower bound of the confidence interval remained higher than the negative of the set equivalence margin *(Δalternative – Δgold standard* > *–equivalence margin)*. Parametric analyses were performed with IBM SPSS statistics 25 software using T-test and confirmed with a 90% CI. We performed non-parametric Mann–Whitney analyses using R-3.6.2 software for data with non-assumed normality. Significance threshold was set at 0.05.

## Results

**Fig. 3:**
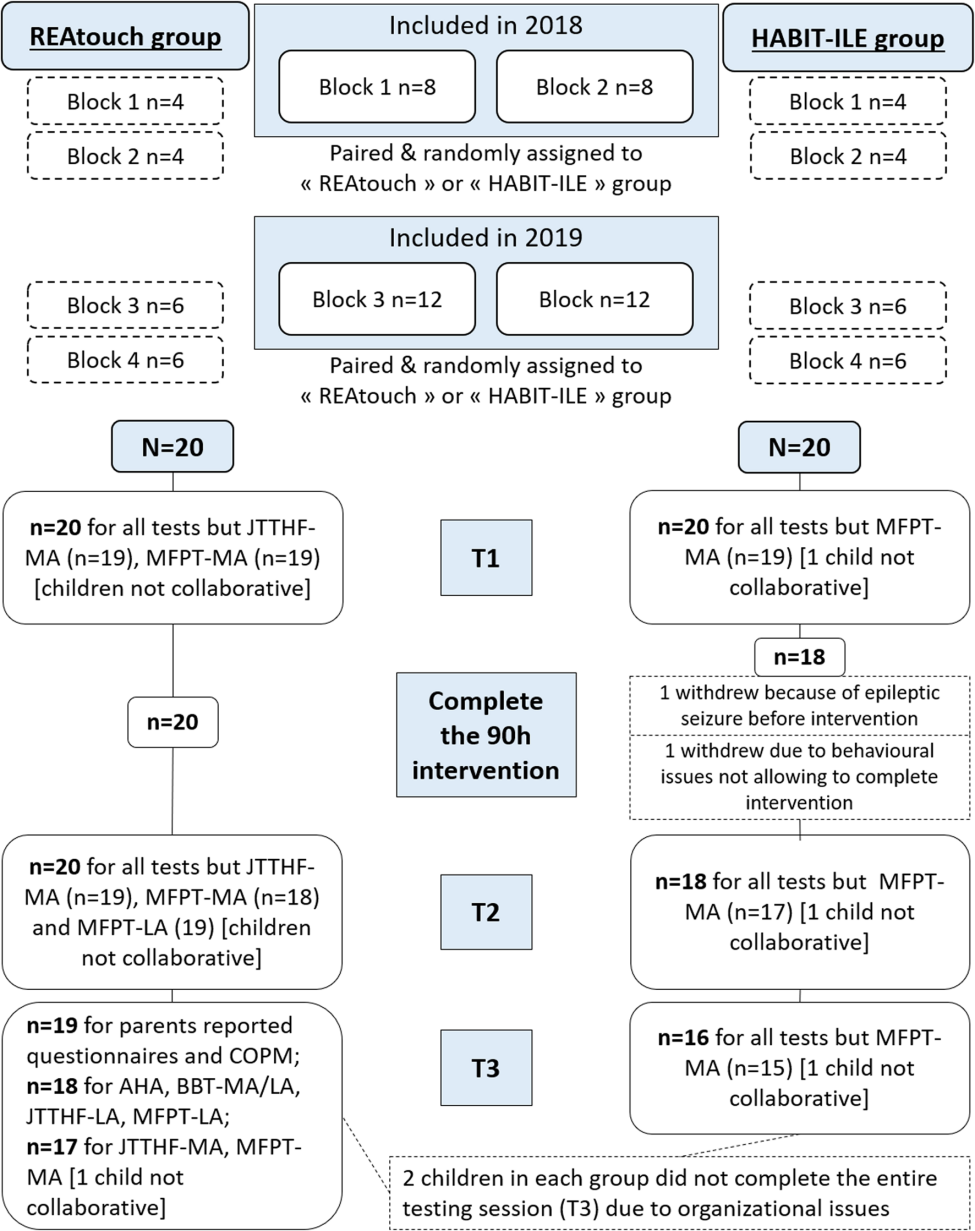
Flow chart of participants. *MA* more-affected hand, *LA* less-affected hand, *AHA* assisting hand assessment, *BBT *box and blocks test, *COPM* Canadian occupational performance measure, *JTTHF* Jebsen-Taylor test of hand function, *MFPT* manual form perception test

Figure 3 displays the flowchart of participants. Among the forty participants, two withdrew in the HABIT-ILE group after the first testing session, and were thus excluded from all analyses. At follow-up, two children in each group had incomplete testing sessions due to organizational issues, leading us to apply intention to treat analysis.

### Treatments characteristics

During the intervention period, as measured through the daily reporting sheets of treatment content, children were on average engaged in play and functional therapeutic activities for 94.6% of the time in the REAtouch^®^ group and 94.9% in the HABIT-ILE group (p = 0.59; Table 2). Children in the REAtouch^®^ group performed REAtouch^®^-based sessions for 40% of the total therapeutic time (41% initially planned; Table 2). Only two significant differences were found between the interventions: a greater percentage of time was spent in standing position in the REAtouch^®^ group (REAtouch^®^ 32%, HABIT-ILE 22%; p = 0.002), and more time was spent in the training of functional activities for the HABIT-ILE group (REAtouch^®^ 25%, HABIT-ILE 33%; p = 0.01; details in Table 2).

**Table 2:** Interventions characteristics based on manually reported activities

	REAtouch^®^	HABIT-ILE	T-tests; Mann-Whitney^a^
Total (% ± SD)	94.6 ± 4	94.9 ± 2	*0.59*^a^
REAtouch (% ± SD)	40.3 ± 3	–	
Upper limb (% ± SD)			
Gross dexterity	56.6 ± 21	51.6 ± 16	*0.42*
Fine dexterity	14.1 ± 17	10.6 ± 10	*0.67*^a^
Card games	0.1 ± 0.4	0.03 ± 0.4	*0.47*^a^
Arts and craft	3.4 ± 3	4.3 ± 4	*0.53*^a^
Functional activities/ADL	25.6 ± 9	33.3 ± 8	0.01*
Other	0	0.01 ± 0.7	*0.31*^a^
Lower limb (% ± SD)			
Ball sitting	51.3 ± 14	57.4 ± 12	*0.07*^a^
Bench sitting	2.5 ± 9	4.4 ± 16	*0.96*^a^
Chair sitting	3.4 ± 3	3.2 ± 2	*0.44*^a^
Standing	32.2 ± 10	22.6 ± 6	0.002*
Transitions	1.4 ± 8	1.5 ± 2	*0.91*^a^
Balance board	0.8 ± 1	0.4 ± 1	*0.35*^a^
Walking/running	2.2 ± 3	2.3 ± 3	*0.84*^a^
Jumping	0.1 ± 0.6	0.4 ± 1	*0.20*^a^
Cycling	4.1 ± 5	6.2 ± 6	*0.33*^a^
Scooter	1 ± 2	0.5 ± 1	*0.55*^a^
Other	0.4 ± 0.7	0.6 ± 2	*0.74*^a^

*ADL* activities of daily living, *SD* standard deviation

^a^Mann–Whitney rank sum test

*Significant results (p < 0.05)

Accelerometer data showed no significant differences between groups, either for the activity duration (75 ± 5% for REAtouch^®^ group and 73 ± 6% for HABIT-ILE group; Mann–Whitney rank sum test p = 0.38) and the mean activity counts (61 ± 17 for REAtouch^®^ group versus 65 ± 16 for HABIT-ILE group; Mann–Whitney rank sum test p = 0.47).

### Motor and functional outcomes

Table 3 displays the observed values, changes during intervention, and non-inferiority statistical analyses for each of the upper and lower extremities outcome measures. While Table 4 shows the results of daily life activities questionnaires and functional goals. One-way RMANOVA showed significant improvements in both groups for the AHA, BBT less-affected hand, JTTHF more-affected hand, ABILHAND-Kids, ACTIVLIM-CP, PEDI, COPM performance and satisfaction measures (all p ≤ 0.039). Only the REAtouch^®^ group showed significant improvements on the JTTHF less-affected hand (p = 0.042). Only the HABIT-ILE group showed significant improvements in the BBT more-affected hand, 6MWT and ABILOCO-Kids (all p ≤ 0.036).

**Table 3:** Upper and lower extremities outcome measures

		Mean ± *SD*			Mean changes ± *SD*		one way RMANOVA^a^	MCID	Non inferiority tests	
									p-value; 90% CI^b^	
		T1	T2	T3	T2–T1	T3–T1	p-value		T2–T1	T3–T1
AHA	REAtouch^®^	54.9 ± 18	58.4 ± 29	56.3 ± 19	3.45 ± 4.4	2.8 ± 3.9	0.002*	5	< 0.001*	< 0.001*
AHA-units	HABIT-ILE	58.3 ± 16	60.3 ± 16	60.6 ± 19	1.9 ± 3.1	1.6 ± 2.8	0.039*		*[− 0.6; 3.6]*	*[− 0.8; 3.2]*
BBT-MA	REAtouch^®^	20.8 ± 13	21.7 ± 13	22.2 ± 13	0.9 ± 4	2 ± 4	*0.092*	1.9	*0.09*	*0.28*
n of blocks	HABIT-ILE	22 ± 11	23.1 ± 11	25.7 ± 15	1.1 ± 3	3 ± 5	0.029*		*[− 2.3; 1.8]*	*[− 3.7; 1.6]*
BBT-LA	REAtouch^®^	45.2 ± 13	47.9 ± 13	51.8 ± 10	2.6 ± 4	5.6 ± 4	< 0.001*	3	0.009*	*0.07*
n of blocks	HABIT-ILE	43.7 ± 15	45.7 ± 16	51 ± 16	2 ± 4	5.8 ± 6	< 0.001*		*[− 1.8; 3.1]*	*[− 3.4; 3]*
JTTHF-MA	REAtouch^®^	419 ± 358	364 ± 333	356 ± 336	− 55.4 ± 87	− 89.3 ± 98	0.018^*,a^	54,7	< 0.001*	< 0.001*
Seconds	HABIT-ILE	412 ± 344	367 ± 344	322 ± 325	− 44.6 ± 113	− 95.2 ± 171	< 0.001*^,a^		*]…; − 21]*^b^	*]…; 34]*^b^
JTTHF-LA	REAtouch^®^	64.6 ± 51	54.2 ± 45	59.1 ± 51	− 10.3 ± 16	− 5.2 ± 21	0.042*^,a^	20,9	< 0.001*	0.002*
Seconds	HABIT-ILE	59.6 ± 48	50.4 ± 26	43.1 ± 16	− 9.2 ± 29	− 13.6 ± 37	*0.174*^a^		*]…; − 19]*^b^	*]…; 6.6]*^b^
MFPT-MA	REAtouch^®^	6.45 ± 3.3	6.7 ± 2.9	6.9 ± 29	0.38 ± 1.7	0.35 ± 1.3	*0.375*^a^	1	< 0.001*	0.042*
Raw score (/10)	HABIT-ILE	6.1 ± 3.1	6.4 ± 3	6.8 ± 3.1	0.29 ± 1.2	0.6 ± 1.4	*0.097*^a^		*[− 0.39; …[*^b^	*[− 0.9; …[*^b^
MFPT-LA	REAtouch^®^	9.65 ± 0.7	9.7 ± 0.5	9.7 ± 1.1	0.0 ± 0.7	0.1 ± 0.6	*0.135*^a^	1	< 0.001*	< 0.001*
Raw score (/10)	HABIT-ILE	9.4 ± 0.7	9.6 ± 0.6	9.5 ± 0.6	0.2 ± 0.8	0.0 ± 08	*0.261*^a^		*[− 0.59; …[*^b^	*[0; …[*^b^
6MWT	REAtouch^®^	467 ± 90	469 ± 101	483 ± 106	1 ± 54	16 ± 57	*0.486*	28.9	0.042*	*0.26*
Meters	HABIT-ILE	478 ± 106	479 ± 117	518 ± 120	1 ± 46	32 ± 58	0.036*		*[− 27; 28]*	*[− 49; 17]*

Outcome results on every testing session and values reported for statistical analyses. Mean values at every testing session are calculated with all the available data, no intent to treat. Mean changes are reported based on data used for non-inferiority analyses, no intent to treat and uncompleted data not included

*SD* standard deviation, *MCID* minimal clinical important difference, *CI* confidence interval, *AHA* Assisting Hand Assessment, *BBT* Box and Blocks test, *JTTHF* Jebsen-Taylor Test of Hand Function, *MFPT* Manual Form Perception Test, *6MWT* 6-Minute Walk Test

^a^Friedmann RMANOVA on ranks

^b^95% CI was calculated for non-parametric Mann-Withney analyses

*Significant results (p < 0.05)

**Table 4:** Daily life activities questionnaires and functional goals

		Mean ± *SD*			Mean changes ± *SD*		One way RMANOVA^a^	MCID	Non inferiority tests	
									p-value; 90% CI^b^	
		T1	T2	T3	T2–T1	T3–T1	p-value		T2–T1	T3–T1
ABILHAND-Kids	REAtouch^®^	1.6 ± 1.8	2.5 ± 2.3	2.4 ± 1.9	0.8 ± 0.9	0.9 ± 0.9	< 0.001*	0.718	*0.18*	0.050*
Logits	HABIT-ILE	1.2 ± 1.4	2.4 ± 1.6	2.18 ± 2	1.25 ± 1.2	0.9 ± 1.4	< 0.001*		*[− 1; 0.2]*	*[− 0.7; 0.67]*
ACTIVLIM-CP	REAtouch^®^	1.9 ± 1.3	2.8 ± 1.8	2.4 ± 1.4	0.97 ± 0.8	0.75 ± 0.5	< 0.001*	0.482	0.016*	0.008*
Logits	HABIT-ILE	1.8 ± 1.1	2.5 ± 1.2	2.4 ± 1.2	0.76 ± 1	0.67 ± 0.7	0.002*		*[− 0.3; 0.7]*	*[− 0.3; 0.4]*
ABILOCO-Kids	REAtouch^®^	2.7 ± 1.7	3.2 ± 1.7	3.3 ± 1.8	0.5 ± 1.3	0.7 ± 1.1	*0.063*	0.753	*0.21*	0.026*
Logits	HABIT-ILE	3.2 ± 1.8	4.1 ± 2.1	3.8 ± 2.1	0.9 ± 1.5	0.5 ± 1.5	0.029*		*[− 1.1; 0.4]*	*[− 0.6; 0.9]*
PEDI	REAtouch^®^	46.5 ± 10	53.7 ± 8	54.1 ± 6	7.2 ± 5	8.3 ± 5	< 0.001*^,a^	3.17	*0.06*	0.047*
Raw score (/63)	HABIT-ILE	47.3 ± 10	54 ± 8	54.1 ± 8	6.6 ± 8	7.6 ± 7	0.004*^,a^		*[− 3.4; 4.4]*	*[− 3; 4.4]*
COPM-P	REAtouch^®^	3.1 ± 1.3	7.6 ± 1	7.1 ± 1	4.4 ± 1.3	4 ± 1.3	< 0.001*^,a^	2	0.001*	0.001*
Raw score (/10)	HABIT-ILE	2.7 ± 0.9	7.7 ± 1	7.2 ± 0.9	4.9 ± 1.3	4.4 ± 1.3	< 0.001*^,a^		*[− 1.2; 0.1]*	*[− 1.2; 0.3]*
COPM-S	REAtouch^®^	3 ± 1.2	8.3 ± 1.3	7.6 ± 1.2	5.2 ± 1.4	4.5 ± 1.3	< 0.001*^,a^	2	< 0.001*	< 0.001*
Raw score (/10)	HABIT-ILE	2.9 ± 1.2	8 ± 0.9	7.6 ± 0.9	5.1 ± 1.4	4.5 ± 1.7	< 0.001*^,a^		*[1; …[*^b^	*[− 1; …[*^b^

Outcome results on every testing sessions and values reported for statistical analyses. Mean values at every testing session are calculated with all the available data, no intent to treat. Mean changes are reported based on data used for non-inferiority analyses, no intent to treat and uncompleted data not included

*SD* standard deviation, *MCID* minimal clinical important difference, *CI* confidence interval, *PEDI* Pediatric Evaluation of Disability Inventory, *COPM* Canadian Occupation Performance measure, *perf* performance measure, *sat* satisfaction measure

^a^Friedmann RMANOVA on ranks

^b^95% CI was calculated for non-parametric Mann-Withney analyses

*Significant results (p < 0.05)

For the parent-reported questionnaires, changes observed in the REAtouch^®^ group showed significant non-inferiority of changes on the ABILHAND-Kids, ACTIVLIM-CP, PEDI, and the ABILOCO-Kids at follow-up testing session (all p ≤ 0.05, details in Table 4). Only the ACTIVLIM-CP showed significant non-inferiority of change at T2 (p = 0.016), with a large variance of changes observed compared to the mean change and equivalence margin in all parents-reported questionnaires. Finally, we observed significant non-inferiority of changes in the performance and satisfaction measures of the fixed functional goals measured with the COPM (all p < 0.001)

The primary outcome (AHA, T3–T1) showed significant non-inferiority of changes in the REAtouch^®^ group compared to the HABIT-ILE group (p < 0.001, details in Table 3). Regarding hand dexterity, results showed significant non-inferiority of changes observed in the REAtouch^®^ group for the JTTHF (both hands, T2–T1 and T3–T1, all p ≤ 0.002) and the BBT less-affected hand (T2–T1, p = 0.009; T3–T1, p = 0.07). Non-significant non-inferiority results were observed for the BBT more-affected hand, with a larger variance of changes observed as compared to the mean change and equivalence margin. Results observed with the MFPT showed significant non-inferiority on both hands (all p ≤ 0.042). For the 6MWT, the changes observed in the REAtouch^®^ showed significant non-inferiority on T2–T1 (p = 0.042), but not for T3–T1 (p = 0.26).

## Discussion

Both groups of children showed significant improvements in most of the outcome measures, much as reported in previous HABIT-ILE interventions for children with unilateral CP [7, 41, 45]. Furthermore, the results showed generally significant non-inferiority of the changes observed in the REAtouch^®^ group compared to the “usual” HABIT-ILE group. Only the BBT and the 6MWT failed to meet the non-inferiority assumption at follow-up assessment.

The results observed in this study suggests the use of REAtouch^®^-based sessions during a HABIT-ILE intervention to be both feasible and effective to improve motor function, with transfer of learning to daily life situations, as highlighted notably by changes in the questionnaire responses. Although feasibility of use was not specifically a matter of inquiry, our findings of no dropouts during therapy in the REAtouch^®^ group tend to indicate general feasibility of its integration within HABIT-ILE day-camps. This is also reinforced by the number of hours performed, which nearly matched the planned percentage of total therapeutic time. However, while feasible and necessary for the present study protocol, the optimal dose of REAtouch^®^ may prove to be less than the present 3–4 h per day. Although this was not systematically measured by questioning children and therapists, REAtouch^®^-based sessions longer than 90 min did sometimes appear to be tiresome for children or therapists. This is in line with literature findings arguing that sustaining long-term client adherence and motivation is a big challenge for virtual-based sessions [16].

Regarding motor and functional outcomes, we argue that the non-inferior changes observed in the REAtouch^®^ group were obtained because, as in a “usual” HABIT-ILE [6] intervention, the key principles for motor skill learning were applied during REAtouch^®^ sessions, namely: intensity of repetitive practice, goal-directed training, shaping/structured activities, self-generated movements driven through adjustment of the affordances of the therapeutic environment (hands off), motivation, and feedback on motor tasks performance.

First, we note that the use of a *hands-off* intervention with self-generated movements was applied in both groups, as no physical guidance or physical help was provided, and the interventionists were instructed and supervised to use only HABIT-ILE therapeutic principles [6]. *Intensity* in terms of repetitive task practice and motor engagement time was monitored with accelerometry data. We expected to observe similar findings in REAtouch^®^ and HABIT-ILE group, as games on the REAtouch^®^ device were designed to stimulate several kinds of object manipulations, repetition of grasps/release, and bimanual coordination, rather than simply moving one object during the game activity. In showing no intensity difference between groups, the results are consistent with previous findings after five days of CIMT showing around 75% of active duration and from 58 to 67 mean activity counts on the more-affected upper limb of children with unilateral CP [46]. Moreover, the present mean activity counts exceeded those in children with unilateral CP (approximately 38.8) playing an AHA session (i.e., activities usually performed bimanually with no specific encouragement from the therapist to use the more-affected hand or shape the objects/activities to the motor abilities) [47]. This emphasizes the role typically played by therapists during HABIT-ILE sessions (with or without the REAtouch^®^) to stimulate the use of the more-affected hand and “shape” the intervention in the performance of challenging but feasible bimanual activities.

In addition to voluntary motor control, motor engagement time and repetitive task practice, one of the main points emphasized in the REAtouch^®^ design was to enable adapting and varying the type of objects and therapeutic environment, with selection of customizable objects to be manipulated, height/tilt of the reactive screen, game difficulty, and active working space area. This flexibility allows therapists to *structure/shape* the intervention based on a given child’s motor abilities and functional goals *(goal-directed)*. Present results for the reported daily activities support the potential of REAtouch^®^-based sessions for structuring the intervention similarly to the usual HABIT-ILE intervention, at least for the upper-extremities; the only significant group differences were the greater percentage of time spent in standing position and a lower proportion of time dedicated to the practice of functional activities in the REAtouch^®^ group compared to the HABIT-ILE group. For the practice of functional activities, the REAtouch^®^-based sessions were mainly directed towards training the motor function abilities needed to perform the functional goals. Some of the goals could be trained directly in the REAtouch^®^ game sessions, for example fasten/unfasten buttons from a tissue and use them as reactive objects, or cutting modelling ingredients with knife and fork when making potions. However, other goals were not amenable for training during the REAtouch^®^-based sessions, which could have contributed to the lower percentage of reported time in, for example, outdoor activities, without interfering in the achievement and retention of functional goals.

*Intrinsic and extrinsic motivation* were, as in the HABIT-ILE intervention, applied through goal-directed training, including purposeful task-oriented practice in play activities conducted in a child friendly environment, with main focus placed on personalized self-determined goals [6]. Motivation during REAtouch^®^-based sessions might well have been enhanced thanks to the engaging nature of interactive virtual systems and the sense of control imparted by self-determined choice among a large variety of games and activities, which included diverse possibilities such as play games, buy and collect artefacts, and “upgrade the avatar” [12, 54].

We find that the REAtouch^®^ system seems to allow the application of motor skill learning principles, as intended. However, the presence of a therapist remains crucial to provide appropriate *feedback* on performance of motor tasks, identify goals, monitor performance, structure the intervention and therapeutic environment, to avoid movement compensations, and to enable the child to transfer the learned skills to their daily life activities [6, 11, 16].

While significant improvements were observed for most of the outcome measures, some did not present non-inferiority for the REAtouch^®^ group. Concerning results on the BBT less-affected hand at follow-up and the parent-reported questionnaires in direct post-camp assessment, we argue the non-significant results may reflect the large variance observed compared to the mean changes and the equivalence margin; here, the upper and lower bounds of the 90% confidence intervals were often extending beyond or lying close to the range of the equivalence margin set. This large variability is explicable by the wide distribution observed for MACS levels (MACS levels: I = 18.4%, II = 50%, III = 31.6%) compared to the data that we used for sample size calculation (MACS levels: I = 12.5%, II = 75%, III = 12.5%) [7].

In considering the non-significant improvements observed for the BBT more affected hand in the REAtouch^®^ group, the similar efficacy of REAtouch^®^-based HABIT-ILE sessions compared to usual HABIT-ILE sessions without the REAtouch^®^ might still be debatable. Although the high rate of motor engagement in bimanual activities seems similar between the sessions with and without the REAtouch^®^, the type of bimanual activities could present some differences. During REAtouch^®^ sessions, a large part of the bimanual activities consists of (dis)assembly an object with the corresponding base to be used on the screen. During such (dis)assembly the role of the more-affected hand consitsmainly in serving as an assisting stabilizing hand. This is potentially less demanding in terms of motor abilities than a task where the child needs to grasp an object directly from the hands of the therapist, with a specific distance, height, orientation, strength, etc.. This hypothesis could account for the potentially lower improvement of hand dexterity of the more-affected hand in REAtouch^®^ sessions compared to usual HABIT-ILE interventions, if we invoke a slightly less specific intervention for part of the performed activities. Nevertheless, the observed results on the AHA, JTTHF and questionnaires concur in showing significant improvements and non-inferiority of change in the REAtouch^®^ group for scores of motor function of the upper extremities and transfer of trained abilities in daily life activities.

For the lower extremities, the results on the 6MWT and ABILOCO-Kids do not allow us to assert the non-inferiority of change in the REAtouch^®^ group compared to usual HABIT-ILE. In certain respects, these results are consistent with those of Chen et al., who showed poorer efficacy of virtual device training on lower extremities motor function of children with unilateral CP compared to bilateral CP [17]. The lesser improvements observed in virtual-based interventions might reflect the complexity of the task of accurately structuring the intervention for lower extremities, since the type of challenging activities for children with unilateral CP could well be difficult to integrate in such environments (e.g., “gross motor and physical activities” in HABIT-ILE; walking on uneven floors, going up/downstairs, riding a bicycle/scooter, or running).

### Strengths and limitations

To our knowledge, this is the first RCT that has tested the non-inferiority of a virtual-based session substituting for nearly one half of the one-on-one therapeutic time in an evidence-based intervention for motor function of the upper and lower extremities in children with CP. Furthermore, as previously recommended, this study documented the therapeutic content and active ingredients of the virtual-based intervention [12]. However, the sample size designed for this study seems to have been too small to highlight clear observations for some of the secondary outcome measures.

Due to limited availability of instrumentation, we had the opportunity to measure accelerometer data on each child for only one therapeutic day. Although the accelerometry results could have shown modifications with a whole-camp monitoring, we assume the results would not be likely to differ based on the obtained results, as well as for the comparison between both groups. Moreover, accelerometers quantify the acceleration of the limb, without differentiation of the type of movement (therapeutically useful or not), nor do they capture fingers/hand fine motoric movements. Measures of activity duration and counts were of interest to document movement intensity and duration, but might not suffice to determine whether movements were indeed task-based activities. Despite its concrete feasibility being questionable, an additional control based on a synchronized video recording could have been carried out to validate whether the measured accelerations indeed corresponded to task-based activities movements.

## Conclusion

Substituting nearly one half of the one-on-one therapeutic time of a high dosage HABIT-ILE intervention with REAtouch^®^-based sessions imparted mainly significant improvements in children with unilateral CP, and non-inferiority of these changes as compared to a “usual” HABIT-ILE intervention in motor function abilities of the upper extremities, transfer in daily life activities and functional goals attainment. In contrast, we observed fewer changes in the REAtouch^®^ group for lower extremities motor function. This is likely due to the difficulty of implementing challenging activities for the lower extremities of children with unilateral CP in such a virtual based therapeutic environment.

### Clinical implications and future research/development

For clinical application, the use of the REAtouch^®^ device seems fit to provide resources that can facilitate therapist decision-making about how best to target the application of motor skill learning principles. To that end, we can recommend a customized training session designed to enhance treatment efficacy and allow the therapist to focus on the patient through an evidence-based approach, without excessive consideration of the device parameters and use [11, 16, 55]. Sessions with the use of REAtouch^®^ based in integration with HABIT-ILE principles seem feasible to improve motor function of the upper extremities and transfer of learning to daily life activities for children with unilateral CP. For motor function of the lower extremities, the potential of REAtouch^®^ sessions would depend on the functional goals, children’s motor abilities, and the opportunities to train lower extremity tasks during virtual sessions. Although our results suggest that REAtouch^®^ based sessions might be more efficient for upper than lower extremities of children with unilateral CP -with possibly greater interest for applying HABIT than HABIT-ILE, we would expect a fundamentally different picture for children with bilateral CP.

Present results should enable the design of future research protocols to further investigate the feasibility and efficacy of implementing the REAtouch^®^ device in other patient populations, notably in children with bilateral CP (as noted above), in different settings with respect to dosage or environment, and perhaps specially to test the potential additional benefits of dedicated training or future developments of the device.

### Supplementary Information

**Additional file 1**: REAtouch^®^ description and use in a HABIT-ILE context.

**Additional file 2**: Illustration of REAtouch^®^-based sessions during HABIT-ILE camp in children with unilateral cerebral palsy.

## Data Availability

The datasets used and/or analyzed during the current study are available from the corresponding author upon reasonable request.

## Abbreviations

6MWT: 6 Minutes walk test
AHA: Assisting hand assessment
BBT: Box and blocks test
CIMT: Constraint induced movement therapy
COPM: Canadian Occupational Performance Measure
CP: Cerebral palsy
GMFCS: Gross Motor Function Classification System
HABIT: Hand-arm bimanual intensive therapy
HABIT-ILE: Hand-arm bimanual intensive therapy including lower extremities
JTTHF: Jebsen-Taylor Test of Hand Function
LE: Lower extremities
MACS: Manual ability classification system
MCID: Minimal clinical important difference
MFPT: Manual form perception test modified by Cooper et al.
PEDI: Pediatric evaluation of disability inventory
UE: Upper extremities
VM: Vector magnitude
