# Correction: Efficacy of integrating a semi-immersive virtual device in the HABIT-ILE intervention for children with unilateral cerebral palsy: a non-inferiority randomized controlled trial

**DOI:** 10.1186/s12984-024-01317-w

**Published:** 2024-02-07

**Authors:** G. Saussez, R. Bailly, R. Araneda, J. Paradis, D. Ebner-Karestinos, A. Klöcker, E. S. Sogbossi, I. Riquelme, S. Brochard, Y. Bleyenheuft

**Affiliations:** 1 UCLouvain, Institute of Neuroscience, COSY Pole, MSL-IN Lab, Brussels, Belgium; 2 Motor Sciences department, FfH Lab, CeREF Santé, HELHa, Rue Trieu Kaisin, 136, 6061 Montignies-Sur-Sambre, Belgium; 3 Fondation Ildys, Brest, France; 4 Laboratoire de Traitement de l’information Médicale (LaTIM), Inserm U1101, Université Bretagne Occidentale, Brest, France; 5 Exercise and Rehabilitation Science Institute, School of Physical Therapy, Faculty of Rehabilitation Science, Universidad Andres Bello, Santiago, Chile; 6 Department of Developmental Neuroscience, IRCCS Fondazione Stella Maris, Pisa, Italy; 7 Haute Ecole Leonard de Vinci, Parnasse-ISEI, Brussels, Belgium; 8 School of Physical Therapy, Faculty of Health Sciences, University of Abomey-Calavi, Cotonou, Benin; 9 Research Institute of Health Sciences (IUNICS-IdISBa), University of the Balearic Islands, Palma, Spain; 10 Department of Nursing and Physiotherapy, University of the Balearic Islands, Palma, Spain

**Correction: Journal of NeuroEngineering and Rehabilitation (2023) 20:98**

10.1186/s12984-023-01218-4

Following publication of the original article [1], supplementary File 2, was originally published without the video link and now it has been corrected.

The original article has been corrected.
