# Supplementary material for: Efficacy of integrating a semi-immersive virtual device in the HABIT-ILE intervention for children with unilateral cerebral palsy: a non-inferiority randomized controlled trial
Source: J Neuroeng Rehabil. 2023 Jul 29;20:98. doi: 10.1186/s12984-023-01218-4 (PMC10385889; doi:10.1186/s12984-023-01218-4)
Supplement: Supplementary file 1 — Additional file 1: REAtouch® description and use in a HABIT-ILE context. [file 12984_2023_1218_MOESM1_ESM.pdf]

Additional file 1 : REAtouch® description and use in a HABIT-ILE context

To demonstrate the use of the REAtouch® device in therapeutic sessions, we present a dedicated video clip in additional file 2.

REAtouch® description

REAtouch® is an interactive medical device designed by Axinesis company (Waver, Belgium, www.axinesis.com) to provide an environment that facilitates decision-making by the therapist about how best to structure the intervention to target the application of motor skill learning principles. The technology was developed in collaboration with clinicians from the Intensive Rehabilitation Foundation (IRF, Brussels), who provided clinical expert guidance throughout the conception and development phase. The device is equipped with a personal computer and a large touch screen, allowing (1) audio-visual and tangible feedbacks to the patient and (2) his/her caregiver to configure appropriately and supervise the rehabilitation with REAtouch®.

More precisely, the REAtouch® system measures 135 cm in length x 75 cm in height and 18.7 cm in length, with a weight of 150 kg. The maximum footprint of the working area is 95 cm in length x 53 cm in width. This workspace can be adapted in view of the patient's morphology and therapeutic objectives (e.g., high/low amplitude movement, stimulation of right or left side, etc.). As shown in the Figure 1, the minimum/maximum

- height of horizontal working area is 57/123 cm.
- inclination of the working area is 0°/85°.
- height of the working area at maximum inclination (85°) is 50.5/117 cm at the bottom edge and 123/187 cm at the top edge.

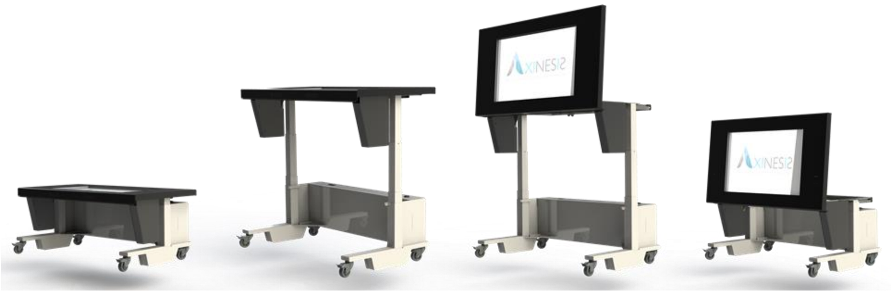

Figure 1. Different configurations of REAtouch®

Saussez, G. *et al.* Efficacy of integrating a semi-immersive virtual device in the HABIT-ILE intervention for children with unilateral cerebral palsy: a non-inferiority randomized controlled trial. *J NeuroEngineering Rehabil* 20, 98 (2023). https://doi.org/10.1186/s12984-023-01218-4

REAtouch® is equipped with the TouchLAB software. TouchLAB allows caregivers to program and manage personalized therapeutic sessions through an intuitive interface (Figure 2).

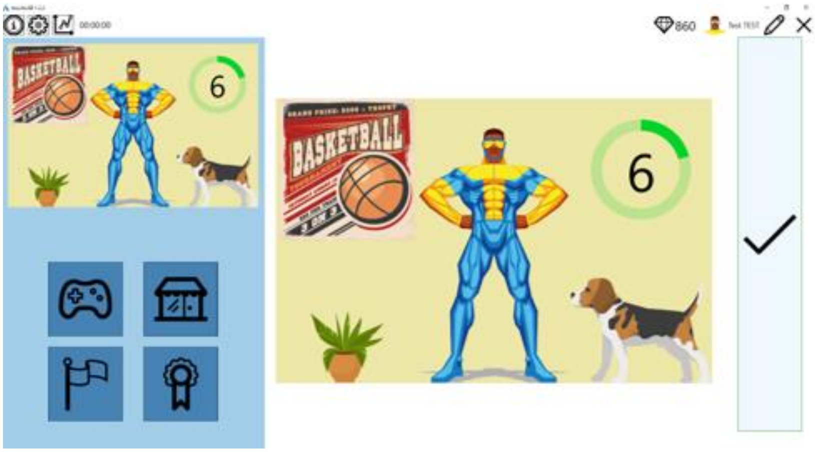

Figure 2. Overview of the TouchLAB software and its patient's personalized interface

TouchLAB software is a personalized interface, such that each user creates his/her own session. By using TouchLAB, the participant can navigate through four main thematic tiles (Figure 3).

The "shop" tile allows the participant to navigate among many different avatar collections, upgrades, and decorations that can be bought with the diamond tokens earned during the games/activities performed with REAtouch®. The participant can use the "flag" tile to set new goals to attain. Those goals can be specific to one therapeutic session or based on specific functional goals sought by the child. A "medal" tile allows the user to see the different medals earned and those yet to earn based on game sessions. Finally, the fourth tile is dedicated to choosing and planning the games/activities to be performed during the session. Depending on the particular game, multiple parameters can also be specified (game difficulty, game duration, working space area, etc).

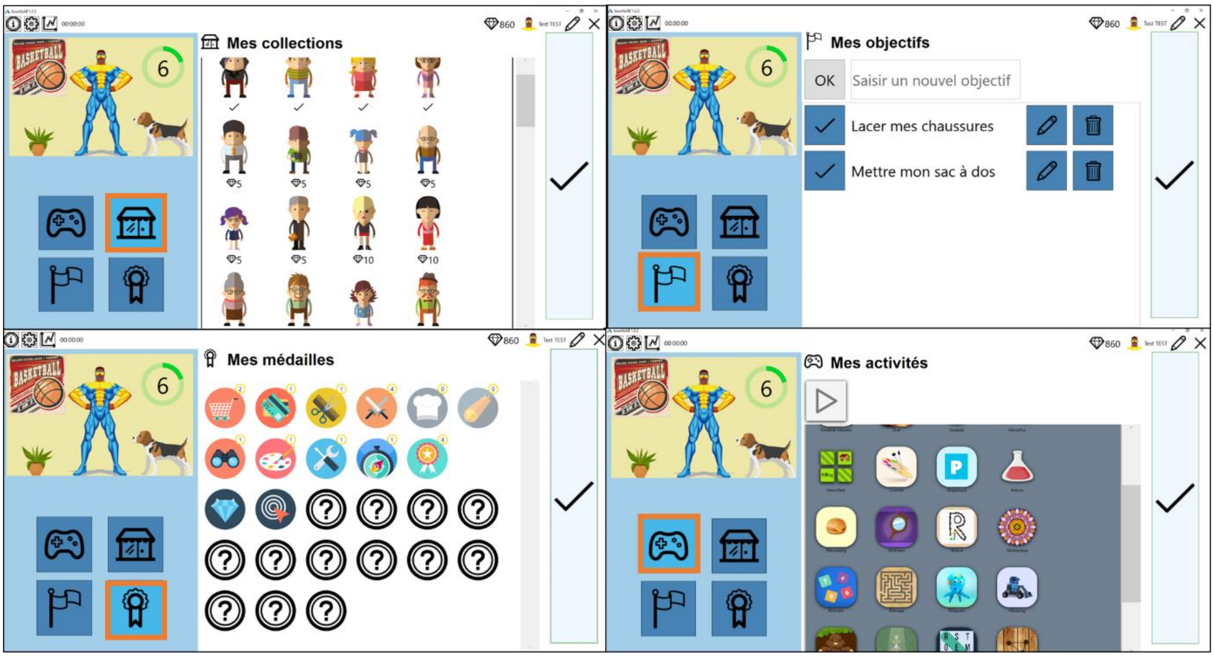

Figure 3. Navigation through TouchLAB software interface

The **upper left** panel shows the “shop” tile. The **upper right** panel illustrate the “flag” tile, which is used to set goals. The **lower left** panel presents the “medal” tile, and the **lower right** panel presents the the “games/activities” tile, which is used to plan the activities to be performed during the session.

Objects-screen interactions

The various types of object-screen interactions can be characterized as “simple contact” and “dedicated bases contact” (Figure 4). Simple contacts can be made by using hand/finger contact or objects on the screen. These contacts are used to navigate in the main part of the TouchLAB interface and in some of the proposed games. For such simple contact interactions, the use of tangible objects is not specifically required, but is recommended as a way to promote object manipulation and bimanual use.

The dedicated bases contacts are used in specific games and activities where the user must fix an object on the desired base and place it on the screen to perform the desired actions. Again, the use of an object fixed on the dedicated base in not absolutely necessary, but is highly recommended to promote manipulation of specific objects. Finally, some of the games/activities in REAtouch® are used with the combination of a dedicated base on one hand and a simple contact interaction for the other hand.

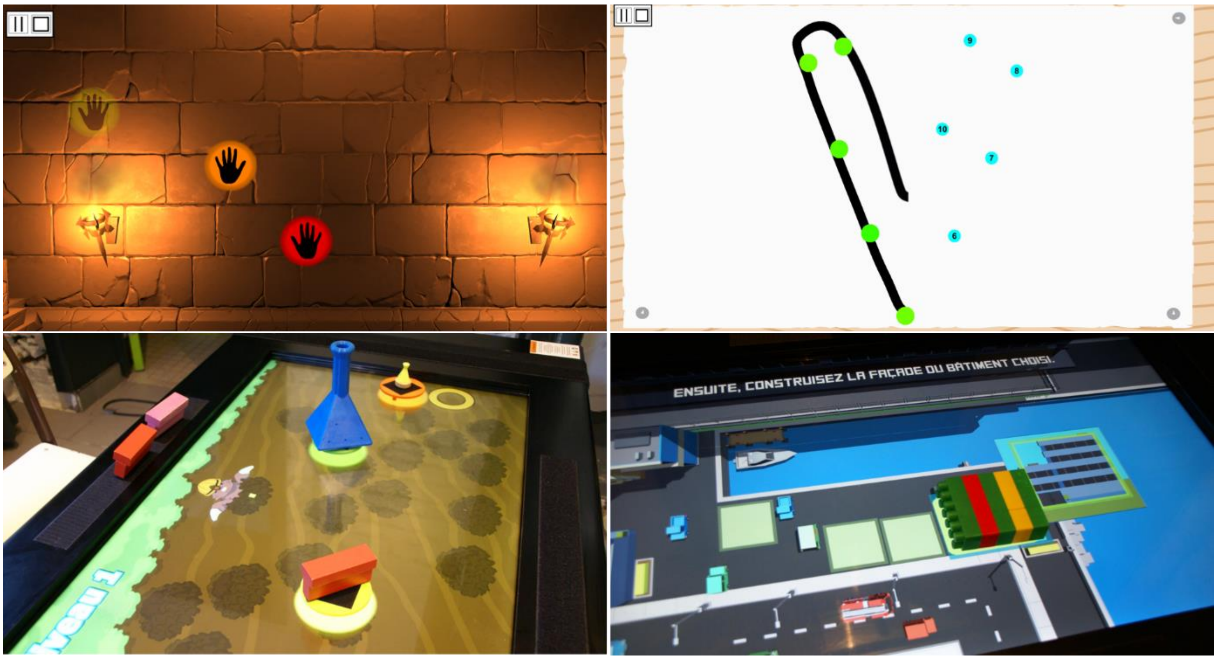

Figure 4. Games and objects-screen interactions

The **left** panels show games with dedicated base interactions, with the **lower left panel** showing objects fixed on the dedicated bases. The **right** panels display games with simple contacts screen-objects interactions, with the **lower right** panel showing object assembly

Use of the REAtouch® in a HABIT-ILE intervention context

In this study, we implemented REAtouch® in the context of a HABIT-ILE (Hand-Arm Bimanual Intensive Therapy Including Lower Extremities) intervention. We now give a detailed description of the use of the REAtouch®, with the objective of applying principles of HABIT-ILE intervention.

HABIT-ILE is a motor skill learning based intervention focusing on the stimulation of bimanual coordination with a constant and simultaneous focus on trunk control and lower extremities (1). The motor learning principles used include intensive practice (high therapeutic dosage and motor engagement time) of game/play activities focusing on personalized, self-determined goals, thus maximizing motivation with a goal-directed training. The training is specific, with an incrementally increasing difficulty of the proposed tasks (shaping) through self-generated movements using affordances of the environment to provide the child with opportunities to find the optimal strategies ("hands off") and with feedback on motor task performance (1). The intervention is

performed in a child-friendly environment with positive reinforcement from the therapist (motivation) (1).

Thanks to specific aspect of its design, the REAtouch® provides resources for clinicians for decision-making about how best to structure the intervention to target the application of motor skill learning principles. This obtained through a constant stimulation of bimanual activities that are fun and motivating, thus affording the possibility of applying the shaping and hands-off principles during functional and goal-oriented activities, notably through the use of a highly adaptable therapeutic environment and the manipulation of real-life tangible objects. However, REAtouch® cannot by itself assume the therapist's roles, which include identifying therapeutic goals, monitoring performance, structuring the intervention and therapeutic environment, and promoting comfortable/transferrable motor patterns, aiming to enable the child to transfer the learned skills to their daily life activities. The presence of a trained therapist or an informed accompanying person under supervision by a trained therapist remains crucial to ensure the correct application of motor learning principles during REAtouch®-based sessions. Table 1 details the application of motor skill learning principles during REAtouch® sessions.

Table 1. Application of the motor learning principles during REAtouch® sessions

	REAtouch®	Therapist
**Intensity of practice**	**+**<br>*Designed for continuously inducing task repetition in a bimanual practice based on screen interactions and object manipulation;*<br>*Opportunities for long term practice with motivating mechanisms (game progression, avatars, goals, medals)*	**±**<br>*Takes advantage of the REAtouch® specific design elements to promote intensity in terms of repetitive task practice and high dosage intervention;*
**Goal-directed training**	**±**<br>*Provides opportunities for the therapist to adapt fully the environment and train a large panel of motor abilities (UE and LE) necessary for functional goals*	**+**<br>*Sets the functional goals with the child and his/her parents;*<br>*Analyzes the functional activities and identifies the ability limitations for each of the goals;*<br>*Determines how to train the ability limitations;*<br>*Chooses how/when to practice goals and promote their transfer in daily life situations*
**Shaping**	**±**<br>*Provides opportunities for the therapist to adapt fully the environment and training of the UE (objects used, games parameters, etc.) and LE (screen height/tilt, etc.)*	**+**<br>*Based on task analysis (see "goal-directed"), the therapist specifically chooses the motor abilities to be trained, prioritizes their training, and adapts intervention to the "just-right" level of difficulty (±75% success rate);*<br>*Monitors performance during the intervention and adapts the tasks according to the trained abilities*
**Hands-off**	**±**<br>*Does not provide any physical guidance*	**+**<br>*Promotes self-generated movements, without physical guidance;*<br>*Adapts the therapeutic environment to train motor functions by using affordances of the environment (e.g. choses objects with different size/weight/texture, gives objects at different heights/orientations or with adapted strength, adapts the height/tilt of the screen, places obstacles on the floor, etc.)*
**Feedbacks on motor task**	**-**<br>*Does not monitor movement*	**+**<br>*Observes the realization of movement and motor performance;*<br>*Provides verbal guidance and/or demonstration to promote motor learning, stimulates a constant bimanual use, and avoids compensations*

Saussez, G. *et al.* Efficacy of integrating a semi-immersive virtual device in the HABIT-ILE intervention for children with unilateral cerebral palsy: a non-inferiority randomized controlled trial. *J NeuroEngineering Rehabil* 20, 98 (2023). https://doi.org/10.1186/s12984-023-01218-4

Table 1. Application of the motor learning principles during REAtouch® sessions *(continued)*

	REAtouch®	Therapist
**Motivation**	+	±
	Includes motivational mechanisms through game activities (auditory/visual reward, gain of diamonds, level of difficulty, etc.);<br>*Provides opportunities for self-determined choices among a large variety of games and activities, which include different possibilities such as play games, buying and collecting artefacts, and upgrading the avatar in a personalized session;*<br>*Goals can be fixed, with reward provided when achieved*	*Takes advantage of the motivational aspects of the REAtouch® to ensure completion of the intervention;*<br>*Constantly provides positive reinforcement with a focus on performance*

+ = actively involved (play an active role for its application); ± = partially involved (allow its use, without playing an active role on it); - = not involved; UE= upper extremities; LE= lower extremities

During REAtouch® based sessions, different type of manipulative/bimanual coordination activities are proposed, based on the different screen objects interactions. Games with a screen interaction with the dedicated bases promote two types of bimanual coordination/manipulation. First, the user must fix/separate the manipulated object and the dedicated base. Second, most of the games are designed to require bimanual simultaneous use with two contact points, one being made by each arm/hand. Real tangible objects are fixed on the bases using Velcro strips, which constitutes a fast and simple way to shape the type of objects manipulation during REAtouch® sessions.

Simple contact interactions are also used for navigating through the interface or in some of the proposed games/activities. Although not always specifically required with this type of interaction, it is recommended that the therapist promotes object manipulation and bimanual use. During HABIT-ILE REAtouch®-based sessions, such simple contact interactions offer opportunities for diversifying the type of manipulations used (dis/assembly of small pieces to use them on the screen, create buildings, finding a path with separated/nested objects, etc.).

As the aim of this study was to integrate the application of HABIT-ILE intervention during REAtouch® sessions, the therapeutic environment had to be adapted and developed accordingly. For stimulating the lower extremities and postural/balance, in

addition to the adaptable height/tilt of the REAtouch® screen, environmental adaptations entail the use of benches, Swiss balls, steps, obstacles on the floor and a standing balance board (for left-right instability). For the upper extremities, a large variety of tangible objects were available to match the particular therapeutic needs of each child. These objects were specially manufactured, derived from usual games/toys, or adapted from daily objects, depending on therapeutic needs (Figure 5). Based on the table published in the methodological paper presenting HABIT-ILE (1), Table 2 describes the type of activities performed during HABIT-ILE sessions using REAtouch® compared to HABIT-ILE sessions without REAtouch®.

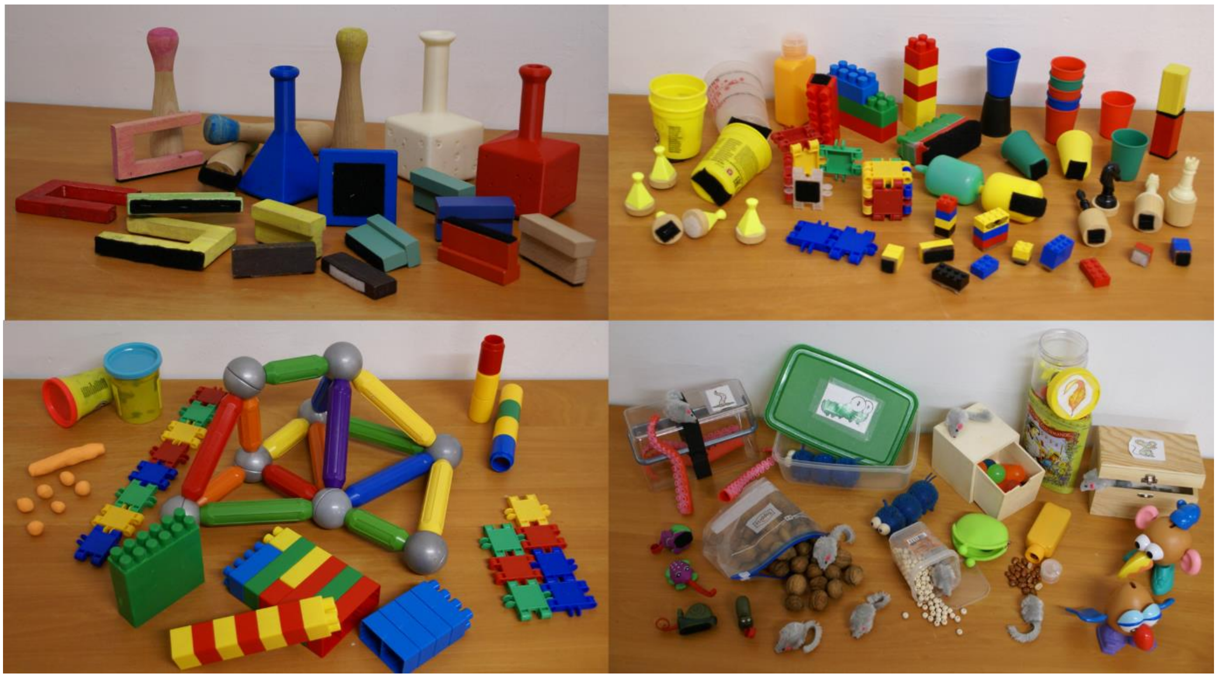

Figure 5. Illustration of therapeutic environment (upper extremities) for REAtouch® sessions based on HABIT-ILE.

The two **upper panels** show objects adapted for use with the dedicated bases (attached by Velcro strips). The **lower panels** display objects and the therapeutic environment dedicated for simple contact screen-objects interactions.

Table 2. Types of activities performed during HABIT-ILE sessions with or without the REAtouch®

	"usual" HABIT-ILE	HABIT-ILE with the REAtouch®
Upper extremities bimanual tasks and activities		
Type of activities	1. Gross dexterity<br>2. Manipulative games and tasks<br>3. Functional tasks<br>4. Art and crafts	1. Gross dexterity<br>2. Manipulative games and tasks
Repetitive task practice	Shoulder flexion/abduction, elbow extension, wrist extension/supination/radial stabilization, types of grasps/precision grasps	
Type of hand use	Stabilizer, manipulator, active/passive assist, symmetrical/asymmetrical movements	
Graded constraints	Type of hand use, spatial and temporal constraints of task	
Lower extremities and trunk control systematically coupled		
Types of activity	1. Sitting on a bench/ball<br>2. Standing (+ transfers)<br>3. Balance board (standing with left-right instability)<br>4. Walking<br>5. Running<br>6. Jumping<br>7. Nordic walking<br>8. Cycling<br>9. Making scooter	1. Sitting on a bench/ball<br>2. Standing (+ transfers)<br>3. Balance board (standing with left-right instability)<br>4. Walking
Repetitive task practice	Bodyweight distribution on both sides, spine straight/aligned, hip flexion/extension/abduction & knee extension, ankle dorsal flexion, symmetry of step length (walking)	
	Symmetry of step length (running), endurance, global balance, increasing step length	/
Type of LE/trunk use	LE stabilizer of balance, constant trunk correction of changes in balance, symmetrical/asymmetrical use of both LE, activities of autonomous movement	
Graded constraints	Gradually ↓ support of the table, ↑ changes in balance (L-R, rotations, etc.), adjust ball inflation, ↑an alternance of standing and squat position, ↓ limitations of standing board movements, changing spatial/temporal constraints of tasks (walking), include obstacles on the floor (walking), concomitantly ↑ difficulty of the task for UE (coordination in standing position)	
	Changing spatial/temporal constraints of tasks (running, jumping, scooter, etc.), include obstacles on the floor (running, jumping, scooter, etc.), concomitantly ↑ difficulty of the task for UE (coordination in walking, jumping, etc.), pass from symmetrical jump to alternate jump, changing the position on the bicycle, changing the supporting foot on the scooter, etc.	/

Table 2. Types of activities performed during HABIT-ILE sessions with or without the REAtouch® *(continued)*

	"usual" HABIT-ILE	HABIT-ILE with the REAtouch®
Practice of functional goals		
Type of activites	All types of functional goals can be practiced (depending on the environment and material available)	Some functional goals can be directly practiced and included into REAtouch® games/activities (e.g., cutting food, opening/closing the zipper of a bag, walking with a tray, etc.).<br>Some other cannot be directly trained during REAtouch® games/activities (e.g., jumping rope, riding a bicycle, tying hairs, etc.)
Graded practice	Goals are initially practice based on the training of motor function/abilities identified as limting for the goal attainment before getting trained in the adapted situation.<br>The training situations gradually evolve towards the real situation of use according to the improvement of the patient's abilities.<br>Finally, transfer of learning is also promoted by practicing in different situations.	When it is possible with the use of REAtouch® device, goals are initially practiced based on the training of motor function/abilities identified as limiting the attainment of a goal.<br>Depending on the goals, some can be trained from adaptation of real situations, with promoting of the transfer of learning.<br>Other goals cannot be directly trained during REAtouch® sessions.

LE = lower extremities; UE: upper extremities; L= left; R= right

Bibliography (Reference)

1. Bleyenheuft Y, Gordon AM. Hand-arm bimanual intensive therapy including lower extremities (HABIT-ILE) for children with cerebral palsy. Physical & occupational therapy in pediatrics. 2014;34(4):390-403.
