# Supplementary material for: Efficacy of integrating a semi-immersive virtual device in the HABIT-ILE intervention for children with unilateral cerebral palsy: a non-inferiority randomized controlled trial
Source: J Neuroeng Rehabil. 2023 Jul 29;20:98. doi: 10.1186/s12984-023-01218-4 (PMC10385889; doi:10.1186/s12984-023-01218-4)
Supplement: Supplementary file 2 — Additional file 2: Illustration of REAtouch®-based sessions during HABIT-ILE camp in children with unilateral cerebral palsy. [file 12984_2023_1218_MOESM2_ESM.pdf]

Additional file 2 (movie): Illustration of REAtouch®-based sessions during HABIT-ILE camp in children with unilateral cerebral palsy

Illustration of REAtouch®-based sessions during HABIT-ILE camp in children with unilateral cerebral palsy, accompanying the article *Efficacy of integrating a semi-immersive virtual device in the HABIT-ILE intervention for children with unilateral cerebral palsy: a non-inferiority randomized controlled trial*, by Saussez et al. in *Journal of Neuroengineering and Rehabilitation*
Link to the video: https://youtu.be/dqfDnlRb6Dg

Description of the above video content:

Situation 1: 0''-1'05''. This segment shows a game entailing the use of simple contact interactions. It illustrates the role of the therapist to promote the use of the more-affected hand in a bimanual activity, with feedback given on the task performance, adaptation of the environment by presenting the objects in a specific position selected to allow the child to train the movements/abilities needed for training of the functional goals.

Situation 2: 1'06''-1'37''. This game includes the use of dedicated bases in interaction with the screen. This video segment displays the role of the therapist in choosing the type of objects for manipulation, promoting the involvement of the more affected hand in a bimanual task adapted to the motor abilities of the child.

Situation 3: 1'38''-2'09''. Use of another game with simple object-screen contact interaction. This video segment illustrates the training of a specific motor ability needed for the achievement of a functional goal. The particular goal was to open chips/biscuits packets. As the child did not at the time of intervention possess sufficient abilities to manage a stable tridigital pinch with the necessary strength, the following motor abilities and strategy were trained; the child practiced the use of the thumb on the more-affected hand (left) to stabilize the paper/packet on the table and use the less-affected hand (right) to tear and open the packet. Training in this motor ability was a notable result of the REAtouch® sessions.

Situation 4: 2'10''-2'29''. This video segment illustrates a game with simple contact interactions and another type of bimanual activity entailing (dis)assembly of the manipulated objects. During this activity, the child directly grasps the objects from the table, which is not usually recommended, as it does not sufficiently enable the therapist to control and modulate the environment in a manner to specifically train the required motor abilities. This demonstration shows that, while the device can be highly useful, it can also be implemented in a non-specific (suboptimal) manner and requires therapists' specificity.

Situation 5: 2'30''-2'47''. The adolescent plays a game entailing the use of dedicated bases. The scenario is similar as in "situation 2". However, due to the child's limited motor abilities of the more-affected hand, the use of that hand in a bimanual activity was engaged only for the (dis)assembly part of the activity.

Saussez, G. *et al.* Efficacy of integrating a semi-immersive virtual device in the HABIT-ILE intervention for children with unilateral cerebral palsy: a non-inferiority randomized controlled trial. *J NeuroEngineering Rehabil* 20, 98 (2023). https://doi.org/10.1186/s12984-023-01218-4
